# The role of P-selectin/PSGL-1 in regulating NETs as a novel mechanism in cerebral ischemic injury

**DOI:** 10.3389/fneur.2024.1442613

**Published:** 2024-07-03

**Authors:** Xiao Li, Yamin Ma, Dongbin Wang

**Affiliations:** ^1^Henan University of Chinese Medicine, Zhengzhou, China; ^2^Nanyang Hospital of Traditional Chinese Medicine, Nanyang, China; ^3^Shenzhen Pingle Orthopedic Hospital (Shenzhen Pingshan Traditional Chinese Medicine Hospital), Shenzhen, China

**Keywords:** neutrophil extracellular traps, NETs, P-selectin, PSGL-1, cerebral ischemic

## Abstract

In recent years, substantial advancements have been made in understanding the pathophysiology of ischemic stroke. Despite these developments, therapeutic options for cerebral ischemia remain limited due to stringent time windows and various contraindications. Consequently, there has been a concentrated effort to elucidate the underlying mechanisms of cerebral ischemic injury. Emerging research indicates that neutrophil extracellular traps (NETs) exacerbate inflammation and damage in ischemic brain tissue, contributing to neuronal cell death. The inhibition of NETs has shown potential in preventing thrombosis and the infiltration of immune cells. Central to the formation of NETs are P-selectin and its ligand, P-selectin glycoprotein ligand-1 (PSGL-1), which represent promising therapeutic targets. This review explores the detrimental impact of P-selectin, PSGL-1, and NETs on cerebral ischemia. Additionally, it delineates the processes by which P-selectin and PSGL-1 stimulate NETs production and provides evidence that blocking these molecules reduces NETs formation. This novel insight highlights a potential therapeutic avenue that warrants further investigation by researchers in the field.

## Graphical abstract

The binding of P-selectin to its receptor PSGL-1 is a key event in the activation of neutrophils. This interaction simultaneously upregulates PAD4 and promotes histone citrullination, leading to NETosis and the release of NETs from neutrophils.

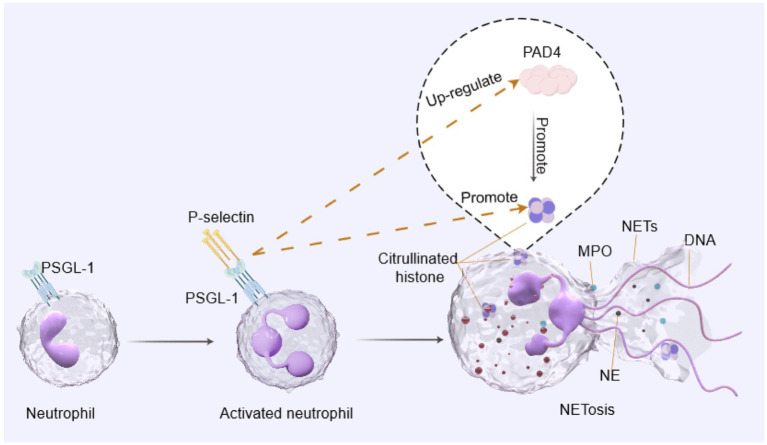

## 1 Introduction

Stroke is the leading cause of mortality worldwide (1, 2) and significantly impacts the daily lives of survivors due to its high incidence of disability. Ischemic stroke, which accounts for approximately 87% of all stroke occurrences (3), is primarily caused by the obstruction of blood supply to the brain. This condition arises from a combination of etiological and related factors. Ischemia triggers a cascade of biochemical events which includes energy depletion, ionic imbalances and excitotoxicity, oxidative stress, cellular death, activation of the complement system, initiation of inflammatory and immune responses, induction of the expression of adhesion molecules on activated endothelial cells, resulting in the rolling of blood-borne inflammatory cells, adhesion and extravasation (4–12). This series of processes ultimately results in permanent damage to the brain (8, 13).

Over the past decade, substantial advancements have been made in understanding the pathophysiology of cerebral ischemia. Despite these advancements, therapeutic options for stroke remain insufficient (11). Pharmacologic thrombolysis or mechanical thrombectomy is recommended for only a small subset of stroke patients, primarily due to time constraints and other contraindications (14, 15). Furthermore, the outcomes of thrombolysis or thrombectomy procedures are not always favorable (16, 17). Consequently, there has been considerable scholarly focus on exploring the pathophysiological mechanisms underlying cerebral ischemic injury, as well as on developing interventions aimed at reducing the incidence of disability and mortality associated with ischemic stroke. Recent evidence suggests that the interplay between inflammation and thrombosis is critical in the pathogenesis of cerebral ischemic injury (18, 19). However, traditional anti-inflammatory and antithrombotic therapies do not adequately address these interactions. This underscores the necessity for the development of innovative therapeutic strategies that specifically target the inflammatory and thrombotic processes involved in cerebral ischemia (18).

It is widely recognized that platelet activation plays a crucial role in the development and progression of thrombosis and inflammation in stroke. P-selectin (CD62p, Selp) is a highly sensitive and specific marker of platelet activation. It facilitates the adhesion functions of platelets, neutrophils, and endothelial cells, thereby initiating thrombosis and promoting inflammatory signaling (20). PSGL-1 serves as the primary ligand for P-selectin and is integral to its various functions (21). P-selectin and PSGL-1 playing pivotal roles in driving NETs formation (14, 22). Recent studies have demonstrated that the formation of neutrophil extracellular traps (NETs) exacerbates inflammation and thrombosis (22), which can have adverse long-term functional consequences after stroke in mice (23). A further study has shown that the presence of NETs in the brains of ischemic stroke patients and the inhibition of NETs represents a potential therapeutic strategy for ischemic stroke (24). Consequently, a more profound comprehension of the role of NETs in brain injury in ischemic stroke, along with the clarification of the underlying mechanisms, may facilitate the identification of novel and promising therapeutic targets. This review will explore how P-selectin and PSGL-1 promote NETs formation, leading to inflammation and thrombosis in patients with ischemic stroke.

## 2 P-selectin/PSGL-1 and NETs

### 2.1 P-selectin and PSGL-1

P-selectin, a 140 kDa granule membrane protein, is part of the selectin family of adhesion molecules (25). Its expression is markedly elevated in the venous blood of patients with progressive ischemic stroke and strongly correlates with the time of disease onset (26). P-selectin is expressed in platelets and stored on the membranes of alpha granules. Upon cell activation, P-selectin translocates to the platelet surface, playing a critical role in the adhesion of activated platelets and monocytes (27, 28). It facilitates leukocyte rolling on post-capillary microvessels, a prerequisite for subsequent leukocyte recruitment to sites of inflammation or infection (20). Consequently, a deficiency in P-selectin results in a delayed recruitment of leukocytes to sites of inflammation. Additionally, the secretion of CCL2 and IL-8 by monocytes critically depends on P-selectin, with CCL2 expression linked to stroke severity (1). CCL2 promotes monocyte mobilization from the bone marrow into the bloodstream by binding to CCR2, implicating the CCL2/CCR2 axis in monocyte recruitment into ischemic brain tissue (1, 29, 30). P-selectin regulates CCL2 expression to control monocyte migration (1).

PSGL-1, a 210 kDa dimeric glycoprotein, binds to P-, E-, and L-selectin (21, 31), facilitating neutrophil aggregation and rolling on endothelial surfaces (32). Its expression is upregulated during inflammation to enhance cell migration into inflamed tissues (21, 33, 34). Upon platelet activation, P-selectin is phosphorylated and translocated to the membrane, where it binds with PSGL-1 (1) (PSGL-1 acts as the primary receptor for P-selectin (35)). This interaction promotes neutrophil recruitment and creates a proinflammatory environment, exacerbating cerebral ischemic injury.

### 2.2 Neutrophil extracellular traps

Neutrophils constitute the primary host defence line against infection and participate in the earliest host defence responses during infection or injury. Neutrophils use various strategies to kill microbes, including phagocytosis, degranulation, production of reactive oxygen species (ROS), production of chemokines and cytokines, and the release of NETs to enhance their anti-microbial properties (36, 37). NETs were first discovered in 1996 (38), Brinkmann et al. (39–42) provide further details of the process and named it NETosis. The release of NETs depends on activation of the NADPH oxidase complex (NOX), which is initiated by the protein kinase C (PKC)-Raf/MERK/ERK cascade (40). PSGL-1 (the primary receptor for P-selectin) has the capacity to activate the ERK pathway (43), thereby facilitating the release of NETs. Furthermore, this complex triggers the activation of enzymes such as myeloperoxidase (MPO), neutrophil elastase (NE), and protein-arginine deaminase type 4 (PAD4) (44–47). PAD4 catalyses histones’ citrullination promoting chromatin depolymerisation and cell lysis. As a result, DNA, citrullinated histones (citH3) and other intracellular particles are released, forming NETs (48–51). A further study has indicated that reducing PAD4 and mitigating the release of NETs may be feasible by suppressing the ERK1/2 signaling pathway (52).

## 3 NETs as a novel mechanism of cerebral ischemic injury

### 3.1 NETs induce thromboinflammation

NETs have the capability to bind to microorganisms, leading to their death or immobilization (41, 53). Additionally, NETs can promote phagocytosis by other neutrophils and phagocytes, contributing to the innate immune response (48, 54, 55). NETs exhibit both beneficial and pathological effects (40, 42, 44, 48). While NETs are believed to primarily capture bacteria and pathogens to combat infections, they exhibit neurotoxicity when formed in the brain parenchyma (56, 57). Excessive NETosis can be detrimental, resulting in uncontrolled inflammatory responses and tissue lesions (40). This process directly causes cell damage and subsequently recruits proinflammatory cells and proteins, forms immune complexes, induces autoantibody production, and causes further tissue damage (40, 58, 59). Moreover, NETs formation can also be stimulated by factors such as cytokines (60) and activated platelets (39). The interaction between neutrophils and platelets leads to the generation of high mobility group protein B1 (HMGB1) (61), which is released by platelets and triggers NETs production. NETs are also thought to stimulate thrombosis (62–64) by cleaving clotting factors, activating platelets, and enhancing thrombin generation, which can result in decreased blood flow and worsen tissue ischemia. This process can cause organ damage by promoting thrombosis and vascular occlusion.

In the context of cerebral ischemia, a complex pathophysiological cascade is initiated, involving both thrombotic and inflammatory pathways, which act as key contributors to ischemic damage (65). Thrombotic and inflammatory processes are highly intertwined factors contributing to cerebral vessel occlusion and stroke, which, in turn, elicits local and systemic inflammatory responses (66). Consequently, novel therapeutic options within the thromboinflammatory field are currently emerging (65). A recent study has demonstrated that post-stroke thromboinflammation may be mediated by pyruvate kinase muscle 2 (PKM2). NETosis exacerbates inflammation and associated damage in ischemic brain tissue (67), and triggers neuronal death (68). Inhibition of NETosis in MCAO animals using PAD inhibitors markedly decreased the infiltration of immune cells and vascular damage (14, 69). In addition, NETs dissolution via DNase substantially decreases BBB injury, augments the coverage of microvascular cells, and improves the formation of new functional blood vessels, consequently reducing thrombosis during ischemic stroke (67, 69, 70). These results indicate that NETs may have a deleterious impact on ischemic stroke. Therefore, Denorme and colleagues concluded that innovative therapeutic interventions targeting the formation of NETs, hold promise as safe treatments for ischemic stroke and should be further investigated (14).

### 3.2 P-selectin/PSGL-1 regulates NETs as a novel mechanism of cerebral ischemic injury

When adhering to leukocytes, activated platelets prompt leukocytes to undergo inflammatory processes (71). Research indicates that activated platelets can induce the formation of neutrophil extracellular traps (NETs) even in the absence of infection, potentially presenting high mobility group box 1 (HMGB1) to neutrophils to facilitate this process (72, 73). HMGB1 plays a critical role in enhancing the production of P-selectin (74), which is essential for the activation of platelets to induce NETs formation (22). P-selectin activates leukocytes through signaling via PSGL-1 (22, 75), promoting the expression of PAD4 and activating the histone citrullination pathway (14, 76), thereby triggering NETosis (22).

*In vitro* (77), P-selectin activates inflammation-related genes in neutrophils via PSGL-1, and the signaling of PSGL-1 promotes the formation of NETs. *In vivo* experiments showed (78) that Antiphospholipid Syndrome (APS) IgG significantly increased thrombosis in WT mice (which did not have PSGL-1 knocked out), while it had no significant impact on PSGL-1 knockouts. Furthermore, the thrombotic phenotype was restored in PSGL-1-deficient mice following the infusion of WT neutrophils, the anti-PSGL-1 monoclonal antibody also inhibited APSIgG-induced thrombosis in WT mice. Further research has demonstrated that the suppression of PSGL-1 leads to a decrease in plasma-based NETs biomarkers, such as myeloperoxidase-DNA, in acute lung inflammation and sepsis animal models (22, 79). Blocking neutrophil PSGL-1 entirely suppressed citrullination of histone H3, as induced by activated platelets. Citrullination of histone is believed to be the most reliable biochemical marker of NETs (80). Thus, these findings propose that the interaction between P-selectin and PSGL-1 activates signals for histone citrullination. This evidence indicates that P-selectin and PSGL-1 have potential as a therapeutic approach to inhibit the formation of NETs and to reduce pathological thrombosis and inflammation (22, 78).

## 4 Conclusion and future directions

In recent years, a growing number of researchers have investigated the role and mechanisms of NETs in disease. NETs have been detected in numerous organ tissues and inflammatory diseases. The approach to managing different ailments is increasingly focused on regulating NETs as a therapeutic objective. The pathogenesis and treatment strategies of neuroinflammation and thrombosis in cerebral ischemia are important areas of research. However, the molecular mechanisms underlying these conditions are not yet fully comprehended by researchers.

Following an episode of cerebral ischemia, it is possible that NETs act as the initial trigger for neuroinflammation and thrombosis. This process is regulated by P-selectin/PSGL-1 and presents a potentially effective therapeutic target for the treatment of cerebral ischemia, using techniques for inhibiting P-selectin or PSGL-1. Nevertheless, the evidence from a murine model of lupus indicates that PSGL-1 deficiency is associated with a reduction in stroke size. However, this is accompanied by an exacerbation of nephritis (81). In light of this limitation, further studies are necessary to assess the mode of action of these inhibitory methods and their impact on the immune system, to select effective treatments devoid of harmful effects.

Our team has long been dedicated to investigating treatments for cerebral ischemic injury (82, 83). Whole transcriptome gene sequencing studies have revealed that numerous inflammatory factors, chemokines, and selectins exhibit differential expression in the brain tissue of MCAO rats compared to control rats. Notably, electroacupuncture treatment significantly modulated the expression of these differentially expressed genes in the brain tissue of MCAO rats (82). Our next objective is to elucidate the biological processes by which P-selectin/PSGL-1 regulates NETs and to conduct a comprehensive investigation into the role and mechanisms of electroacupuncture in mitigating cerebral ischemic damage. We anticipate that in the future, targeting P-selectin/PSGL-1 or NETs will have broad applicability in the treatment of cerebral ischemia.

## Author contributions

XL: Conceptualization, Writing – original draft. YM: Writing – review & editing. DW: Supervision, Writing – review & editing.
